# The American Medical Association

**Published:** 1882-07

**Authors:** 


### Editorial.

### The American Medical Association.

The thirty-third meeting of the American Medical Association was convened at St. Paul, Minn., on the 6th of June last and continued in session four consecutive days, its transactions being of an exceedingly interesting character; medical men, whose names alone insured an attentive audience, being attracted there from all sections of the country, while the younger, and less known, members of the profession were largely represented. The limited space accorded by the Journal to this report precludes a detailing of each subject brought up for discussion in the Society, and, indeed, such is rendered a work of supererogation by the full accounts of the proceedings given daily in the public press; a mere synopsis is therefore here presented touching only on a few of the salient points.

The Association was called to order at 11 A. M. on the 6th inst., a concourse of spectators of both sexes, besides the delegates, crowding the large Opera House of the city to overflowing, their extreme interest causing them to throng the stairs and select seats in the parquette and galleries long before the opening hour.

At the appointed time Chairman Stone conducted upon the stage Dr. P. C. Hooper, Gov. Hubbard and Bishop Ireland, and the latter inaugurated the convention by an appropriate prayer, according to the universal custom of such assemblages.

36

At its conclusion Gov. Hubbard was introduced, who welcomed the distinguished strangers with much cordiality and warmth. He said the people of his State felt highly honored by the presence of such a body of gentlemen, and he trusted the visit would not terminate with the completion of official duties alone. To physicians, Minnesota affords peculiar interest and invites especial investigation, as the exhilarating and vitalizing climate have made it a Mecca for health-seekers in past years, and he expected its reputation, in this respect, would be greatly widened and strengthened by a personal knowledge of its advantages. It was not, however, his purpose now to extol these, but rather to greet those present in the name of the people and assure them of their interest and sympathy in the coming deliberations.

The Vice-Presidents and ex-Presidents were then invited to occupy seats upon the platform. Dr. Sayre, of New York; Dr. Davis, of Chicago; Dr. Toner, of Washington, and Dr. Cole, of California, being hailed with applause as they ascended to their places. Immediately following this the ethical question was agitated; the Secretary announced that numerous protests had been received against the admission of delegates from the New York Medical Society, and condemning its course in reference to the Code of Ethics, deeming it an insult to the Association. The utmost indignation at the action of the recalcitrants was evidently felt and expressed in all parts of the Union. The letters of Drs. Sayre, of New York, and Gross, of Philadelphia, were also read, being greeted by a furor of applause, and the protests were then referred to the Judicial Council.

When the excitement, occasioned by the reading of the protests had subsided, Dr. Stone verbally presented, on behalf of the Committee on Arrangements, a programme of railroad excursions, receptions and entertainments by private individuals, and a banquet by the profession and citizens at the Metropolitan Hotel. Notably among the excursions was one to Stillwater and to the White Bear Lake.

These hospitable intentions being announced and disposed of, the annual address was delivered by the 1st Vice-President, Dr. Hooper; the President—Dr. J. J. Woodward, U. S. Army—having been obliged to go abroad on account of ill-health.

After a regretful reference to the sad reasons causing the absence of his distinguished colleague, and a complimentary mention of his predecessors in office, Dr. Hooper discharged his first duty by a grateful acknowledgment of the hearty reception extended by the citizens of St. Paul. He then sketched the history of the association, its aims and success, and in connection with its age, alluded briefly to those of its founders who had passed away since its inauguration, paying a tribute also to the few honored survivors. The retrospect over past years of science in general naturally suggested to him the contrast between the derision with which Newton's views were met by the public, and the liberality now accorded to each new idea. To-day, he said, every thinker has substantial recognition; the people are being educated continually by schools, libraries, lectures, etc. "The skepticism of the age is not afraid of the new and startling; the tendencies are to prove all things, holding fast that which is good. The cry of Sir Thomas Bodley to Bacon 'a fresh creating new principles of science would be to be disposed of the learning we have' is not needed." He noted the progress which had been made by medical science within the last year, and dwelt upon the important influence the Association would exert in reforming medical education. He believed this influence would be further increased by the substitution of a weekly medical journal in lieu of the present annual volume of transactions. He earnestly called attention to the recent action of the New York Medical Society, and in concluding this topic remarked: "One mistaken movement would involve us in a whirl of inconsistencies, tending to place us in a false attitude and bring dishonor upon the profession. The broad lines of demarcation between the irregular and the true physician should never be obliterated. Our Association stands prominently forth in its high purposes, and its means of accomplishing these purposes are distinctly enunciated."

Finally he glowingly eulogized the President of last year, Dr. J. T. Hodgen, whose death occurred at St. Louis but a few weeks since.

The address was followed by the transaction of some small matters of business, and the presentation of a series of resolutions from the Women's National Christian Temperance Union by Dr. Davis, of Chicago, who felt, in offering them, that the universal woman heart beat in unison with the sentiments therein expressed.

The Section on Medical Practice assembled in the afternoon of the first day, the subject presented being: The Practice of Medicine, Materia Medica and Physiology. The Section was called to order by Dr. John A. Octerlony, of Louisville, who later read the address in medicine. The reading of the first paper, on Therapeutic Action of Chlorate of Potassium, by Dr. J. V. Shoemaker (Philadelphia), was postponed till after that of Dr. J. H. Tyndale on Home Treatment of Pulmonary Consumption by General Local Antisepsis on the Basis of Strict Individualization.

Dr. Donnelly, of New York, addressed the Section on the Use of Salicylate of Potassa in Acute Rheumatism and Dyspepsia. The merits of the subject were afterwards fully discussed, and the paper referred to the Committee on Publication.

The Section on Surgery and Anatomy also convened in the afternoon with a large attendance, and was called to order by Dr. Wm. A. Byrd, of Quincy, Ill., the Secretary, who was subsequently elected Chairman, and Dr. McCall, of Michigan, Secretary. Several papers of interest were presented, and in place of one passed, a paper on Anchylosis of the Hip, by Dr. C. C. F. Gay, of Buffalo, was read. He distinguished between true and false anchylosis, giving the false as the rule, and the true as the exception. He said no age is exempt from it; movable articulations show both forms and anæsthesia is often necessary to determine the true and the false. An illustrative case was cited from his service at the Buffalo General Hospital, where an operation was performed with good prospect of success. The paper was discussed at length.

A contribution to the Surgery of the Liver was offered by Dr. Joseph Ranoshoff, of Cincinnati, who reported two important cases successfully operated upon by novel methods. Another paper was on The proper Points for Incision in the Drainage of Suppurating Knee Joints, by Dr. Andrews, of Chicago, which paper was prefaced by the expression of the opinion that the reading of all the papers (and afterwards discussing those desired) was the correct way and should have been earlier adopted.

At the meeting of the Section on Ophthalmology, Otology and Laryngology the election of officers was as follows: President, Dr. Jones, of Chicago; Secretary, Dr. Carl Seiler, of Philadelphia. Valuable information was given and interesting cases cited by Drs. Agnew, of New York; Seiler, of Philadelphia; Calhoun, of Atlanta, and other prominent specialists. The matter of communicable diseases of the eye received considerable attention.

The State Medicine Section was called to order by Dr. A. L. Gibson, U. S. N., and proceeded immediately to discuss the questions referred to them by the Association.

The branch of the profession interested in Dentistry held its meeting under the organization of the Chairman of last year, Dr. D. H. Goodwille, of New York; subsequently electing him President, and Dr. T. W. Brophy, of Chicago, Secretary.

The report of the Committee on Journalizing the Transactions of the Association was presented by Dr. Packard, of Philadelphia, and it was resolved that a Board of Trustees be appointed to agree, as early as possible, upon a plan of a medical journal, to be called the *Journal of the American Medical Association;* said Board to send circulars to all members of the profession throughout the entire country, asking pledges of support and ascertaining, reliably, what basis the proposed journal can have for commencing publication. Suggestions were added in regard to the editor's salary, subscription price, etc.

Following this Dr. Charles Denison, of Denver, introduced a resolution to the effect:

"That, in order to correct a misconception existing in the public mind, and also prevailing among the medical profession, as to the liberty authorized by the Association in the treatment of disease, a declaration of principles, as sanctioned under our code, was deemed proper, namely: Rational medicine demands absolute freedom in the methods of administration of materia medica, and nothing in our code of ethics prohibits the use of any known and honorable means of combating disease. We hail with pleasure and gratitude every discovery in therapeutical science, by whomsoever made. We therefore reject as obnoxious the term "allopathists." First—because it tends to convey the erroneous impression that we are restricted in our choice of remedies. Second—because we have always held it dangerous in practice and unscientific in principle for any association of men, claiming to be physicians, to adopt a name based upon limited theories of therapeutics, for the purpose of designating a particular school of medicine."

The report of the Librarian, Dr. Wm. Lee, of Washington, included a full list of books added to the library during the year. This addition makes the library consist at present of 1,702 distinct titles, which comprehend from a general estimate about 4,448 volumes, inclusive of pamphlets. The Boston Medical Library Association has generously placed a large number of its duplicate periodicals at the disposal of this library, which have gone far towards completing certain imperfect sets of the same.

The report of the Treasurer, Dr. R. J. Dunglison, was read, showing a balance of $1141.38 in the treasury at this date.

Dr. Keller, Arkansas, offered for action the resolution:

"That in many of our large cities cremation will soon become a sanitary necessity."

Dr. J. G. Thomas, of Georgia, presented the following resolution on medical examinations:

"*Resolved*, That the Association approve the organization of Faculties in medicine having no other foundation than the examination for degrees as a measure which will increase the value of the present methods of education in medical colleges in this country."

A motion to lay on the table was lost, but after some further discussion the subject was postponed.

EVERY OTHER MEETING TO BE HELD IN WASHINGTON.

N. S. Davis introduced the following:

"*Resolved*, That after the next annual meeting the permanent interests and influence of this Association would be best promoted by again holding every second meeting in Washington, as its home on common national ground and not as invited guests, while each alternate meeting should be held in such section of the Union as would be most useful in promoting the society organizations in all parts of our country." Adopted.

The Committee on Nominations presented their report and the following is the list of officers elected for the ensuing year:

President—Dr. John L. Atlee, Philadelphia.

First Vice President—Dr. Eugene Grissom, North Carolina.

Second Vice President—Dr. A. J. Stone, Minnesota.

Third Vice President—Dr. J. A. Octerlony, Kentucky.

Fourth Vice President—Dr. H. S. Orme, California.

Treasurer—R. J. Dungleson, Pennsylvania.

Librarian—Wm. Lee, Washington.

Members of Judicial Council—N. S. Davis, Illinois; J. M. Brown, United States Navy; X. C. Scott, Ohio; M. Sexton, Indiana; N. C. Husted, New York; Wm. Lee, Maryland; J. E. Rives, West Virginia.

OFFICERS OF THE VARIOUS SECTIONS.

Practice of Medicine—J. H. Hollister, Illinois, Chairman; J. G. Lee, Pennsylvania, Secretary.

Surgery and Anatomy—W. F. Peck, Iowa, Chairman; Paul F. Eve, Tennessee, Secretary.

Obstetrics—J. K. Bartlett, Wisconsin, Chairman; G. A. Moses, Missouri, Secretary.

Medical Jurisprudence and State Medicine—Foster Pratt, Michigan, Chairman; Thos. L. Neal, Ohio, Secretary.

Ophthalmology, Otology and Laryngology—A. W. Calhoun, Georgia, Chairman; Carl Seiler, Pennsylvania, Secretary.

Diseases of Children—R. Blount, Indiana, Chairman; J. H. Sears, Texas, Secretary.

Dentistry—D. H. Goodwillie, New York, Chairman; T. W. Brophy, Illinois, Secretary.

The Committee further announced that Cleveland, Ohio, had been selected as the place of meeting for 1883. The President elect, Dr. John L. Atlee, was then escorted to the platform and made a brief address. Thanks were tendered to the retiring President, Dr. P. O. Hooper, and the Association adjourned to assemble at Cleveland on the first Tuesday in June, 1883.

